# Variation of iron redox kinetics and its relation with molecular composition of standard humic substances at circumneutral pH

**DOI:** 10.1371/journal.pone.0176484

**Published:** 2017-04-28

**Authors:** Ying Ping Lee, Manabu Fujii, Tetsuro Kikuchi, Koumei Terao, Chihiro Yoshimura

**Affiliations:** 1 Department of Civil and Environmental Engineering, School of Environment and Society, Tokyo Institute of Technology, Ookayama, Meguro-ku, Tokyo, Japan; 2 Ibaraki Kasumigaura Environmental Science Center, Okijyuku-machi, Tsuchiura, Ibaraki, Japan; VIT University, INDIA

## Abstract

Oxidation and reduction kinetics of iron (Fe) and proportion of steady-state Fe(II) concentration relative to total dissolved Fe (steady-state Fe(II) fraction) were investigated in the presence of various types of standard humic substances (HS) with particular emphasis on the photochemical and thermal reduction of Fe(III) and oxidation of Fe(II) by dissolved oxygen (O_2_) and hydrogen peroxide (H_2_O_2_) at circumneutral pH (pH 7–8). Rates of Fe(III) reduction were spectrophotometrically determined by a ferrozine method under the simulated sunlight and dark conditions, whereas rates of Fe(II) oxidation were examined in air-saturated solution using luminol chemiluminescence technique. The reduction and oxidation rate constants were determined to substantially vary depending on the type of HS. For example, the first-order rate constants varied by up to 10-fold for photochemical reduction and 7-fold for thermal reduction. The degree of variation in Fe(II) oxidation was larger for the H_2_O_2_-mediated reaction compared to the O_2_-mediated reaction (e.g., 15- and 3-fold changes for the former and latter reactions, respectively, at pH 8). The steady-state Fe(II) fraction under the simulated sunlight indicated that the Fe(II) fraction varies by up to 12-fold. The correlation analysis indicated that variation of Fe(II) oxidation is significantly associated with aliphatic content of HS, suggesting that Fe(II) complexation by aliphatic components accelerates Fe(II) oxidation. The reduction rate constant and steady-state Fe(II) fractions in the presence of sunlight had relatively strong positive relations with free radical content of HS, possibly due to the reductive property of radical semiquinone in HS. Overall, the findings in this study indicated that the Fe reduction and oxidation kinetics and resultant Fe(II) formation are substantially influenced by chemical properties of HS.

## Introduction

Iron (Fe) is an essential micronutrient for various metabolic processes of organisms such as photosynthesis, respiration, processing of intracellular reactive oxygen species and nutrient acquisition [1]. Previous studies over the last few decades have shown that the bioavailability of Fe in natural waters is tightly regulated by Fe redox transformation kinetics and resulting chemical speciation, which can be affected by the physicochemical factors such as light, pH, reactive oxygen species (ROS), dissolved oxygen (O_2_) and organic and inorganic ligands [2,3]. Under the circumneutral pH and air-saturated conditions (e.g., euphotic zones of oceans and lakes), ferrous iron (Fe(II)) is rapidly oxidized to thermodynamically stable ferric iron (Fe(III)) by oxidants such as O_2_ and hydrogen peroxide (H_2_O_2_) [4–7]. For example, inorganic Fe(II) can be oxidized with half-life on the order of minutes in seawater (pH ~8.0 and temperature of 25°C) [5–8]. While O_2_ is likely a major oxidant for Fe(II) in natural surface waters containing Fe(II) at nanomolar concentrations, H_2_O_2_ can be significantly involved in Fe(II) oxidation when present at relatively high concentrations (e.g., several hundred nanomolar or more) [9–11]. Inorganic Fe(III) is readily removed from the solution phase by precipitation of hydrolysis species, resulting in extremely low concentration of dissolved inorganic Fe at circumneutral pH (e.g., ~10^−11^ M at pH 7.5–9, [12]). Due to the low Fe(III) solubility and external input such as atmospheric dust deposition, concentrations of dissolved Fe in surface waters of the remote open oceans are as low as 0.03–1.0 nM [13]. Therefore, Fe is a limiting factor for the primary production in one-third of the world ocean where macronutrient concentrations are perennially high [4].

The presence of natural organic matters (NOM) including humic substances, however, increases the Fe solubility by up to several orders of magnitude compared with ligand-free medium due to the formation of organically complexed Fe [12,14]. Concentrations of dissolved Fe in freshwater and coastal waters (e.g., ~1 nM–~10 μM) are relatively high due to the complexation by NOM with terrestrial origin [15]. The complexation of Fe by NOM can be affected by several factors such as pH, competitive cations, NOM concentration and possibly type of NOM [16]. Previous study by Fujii et al. [17] indicated that Fe complexation by HS such as Fe-binding capacity can be affected by molecular composition of HS (e.g., aromaticity), suggesting that Fe complexation occurs in a different manner depending on the types and origins of HS. In addition, the Fe(II) oxidation reaction by dissolved oxygen at circumneutral pH is also influenced by characteristics (e.g., aromaticity) and origin (microbial and terrestrial sources) of NOM [18–20].

It is recognized that Fe(II) formation is of importance for Fe bioavailablity in natural waters, since Fe(II) is more soluble at circumneutral pH and available for uptake by microorganisms including phytoplankton [3]. The generation of thermodynamically unstable Fe(II) in surface natural waters is in many cases attributed to the thermal (dark) and photochemical reduction of organically complexed Fe(III) via reactions with redox-active moieties (e.g., quinone-hydroquinone) of HS, light-induced ligand-to-metal charge transfer (LMCT) reactions and superoxide- and microbially mediated processes [21–25]. Borman et al. [26] indicated that thermal reduction of Fe(III) in a mountain river containing high concentration of dissolved organic carbon is the important process in the formation of the quasi-steady-state Fe(II). The thermal reduction of Fe(III) in the presence of a soil humic acid or fractionated NOM (polyphenolic-rich or carbohydrate-rich fractions) was found to be correlated with the contents of aromatics at pH > 4 under the anoxic condition [21]. The presence of HS with a higher content of carboxyl functional groups also increases the biologically-mediated reduction rate for Fe(III) minerals in soil [27–29]. These studies indicated that the Fe(III) reduction can be enhanced due to the increased Fe(III) complexation capacity of NOM, given the carboxyl group is a major Fe-binding site in NOM. Besides carboxyl content, the rate constants for thermal reduction of Fe(III) complexed by Suwanee River fulvic acid (SRFA) were found to decrease with increasing solution pH most likely due to the higher degree of competition with Fe(III) precipitation at higher pH [22].

In addition to the thermal reduction, photochemical reactions in euphotic zones induce transient formation of Fe(II) particularly when NOM is present as a chromophore [30]. The LMCT reactions and superoxide-mediated processes are likely the major mechanisms in the photochemical Fe(II) generation from Fe(III) complexes by NOM [24,25]. For example, a substantial decrease of O_2_ consumption after the methylation of carboxyl groups of aquatic HS in colored surface waters suggests that O_2_ consumption occurs via the charge-transfer mechanism and concurrent photochemical reduction of Fe(III) coordinated with carboxyl groups [31,32]. The presence of organic ligands that outcompetes Fe(III) precipitation via formation of organically complexed Fe(III) (instead of Fe(III) hydroxide complexes) fundamentally alters the photo-reduction pathways of Fe(III) [33]. Rijkenberg et al. [34] examined the effect of model Fe-binding ligands (i.e., tetrapyrrole ligands [phaeophytin and protoporphyrin IX], trihydroxamate siderophores [ferrichrome and desferrioxamine B] and an inositol hexaphosphate [phytic acid]) on the Fe(II) photo-production using seawater of the Southern Ocean, and found that the Fe(III) photo-production rates depend on the Fe-binding ligands used possibly due to the different degree of stability for Fe(II) and Fe(III) complexes. Meunier et al. [35] also reported that the low-molecular-weight NOM fractions (<1 kDa) produced higher steady-state Fe(II) fractions (concentration of steady-state Fe(II) relative to total dissolved Fe concentration; i.e., [Fe(II)]_ss_/[Fe]_T_) compared to the high-molecular-weight fractions (1 kDa < NOM < 0.22 or 0.45 μm) in both the irradiated freshwater and seawater samples.

The previous studies on thermal and photochemical reductions suggest that the complexation of Fe(III) by NOM is one of the important factors in the formation of Fe(II) at discernible concentration in surface natural waters [35,36]. In addition, composition and source of NOM are likely other important factors in Fe(II) generation from Fe(III)-NOM complexes. However, the underlying mechanisms in the Fe(II) formation have yet been fully elucidated particularly in relation to the effect of NOM characteristics and compositions. Since the Fe redox kinetics are associated with stability of ferrous and ferric iron complexed with ligands as well as reducing capacity of NOM [37], the Fe redox reactions are expected to be affected by the chemical properties of NOM. In this study, therefore, we investigated the rates of Fe(II) oxidation by O_2_ and H_2_O_2_ as well as Fe(III) photochemical and thermal reduction at circumneutral pH (pH 7.0–8.0) in the presence of chemically well-defined standard HS with various origins. The experiments in this study were conducted at high HS: Fe ratio to ensure that Fe(III) precipitation does not significantly complete with redox reactions. Based on the experimental results, the relations of Fe redox rate constants and [Fe(II)]_ss_/[Fe]_T_ to the chemical properties of HS were discussed. Given that Fe(II) is a major substrate for uptake by microorganisms in natural waters, it is important to investigate the effects of NOM characteristics on the quasi-steady-state concentration of Fe(II), which can be dynamically regulated by the balance of redox reactions of Fe and NOM complexes.

## Materials and methods

### Reagent preparation

All the reagents and solutions were prepared by using purified water (W-20, Trusco Nakayama Corporation, Japan) and stored in the dark at 4°C when not in use. In order to avoid contaminations by trace metals, glassware was soaked in 5% nitric acid bath (HNO_3_, Kanto Chemical, Japan) overnight, followed by rinsing with purified water prior to use. Adjustment of solution pH was performed by titration of 1–10 M hydrochloric acid (HCl, Kanto Chemical) and sodium hydroxide (NaOH, Kanto Chemical). Measurement of pH was performed by using a pH meter calibrated by JIS calibration buffers (HM-25R, TOA DKK, Japan). Prior to the experiments, water temperature of solution (e.g., reagent and sample) was adjusted to 25°C by using a water bath. All laboratory measurements were performed in a temperature-controlled room at 25°C.

The stock solutions of standard HS were prepared by dissolution in 0.1 M NaOH (reagent grade, Kanto Chemical) at the final concentration of 10 g L^-1^, followed by pH adjustment to 8.0 ± 0.05 using 1–10 M HCl. Standard HS were purchased from International Humic Substance Society (IHSS) (i.e., Suwannee River II humic acid [2S101H], Elliott Soil humic acid [1S102H], Pahokee Peat humic acid [1S103H], Leonardite humic acid [1S104H], Nordic Lake humic acid [1R105H], Waskish Peat humic acid [1R107H], Suwannee River I fulvic acid [1S101F], Suwannee River II fulvic acid [2S101F], Nordic Lake fulvic acid [1R105F], and Pony Lake fulvic acid [1R109F]) and Japanese Humic Substance Society (JHSS) (i.e., Dando Soil humic acid [DHA], Inogashira Soil humic acid [IHA], Inogashira Soil fulvic acid [IFA] and Biwa Lake fulvic acid [BFA]). Chemical properties of BFA [38], IFA and IHA [39], DFA and DHA [39], and other HS from IHSS [40] were obtained from the literature. The origin and fundamental chemical properties including elemental compositions, carbon species and contents of acid functional groups are well-defined for these standard HS, as listed in S1 Table.

In the Fe(II) oxidation experiment, a 4.0 mM Fe(II) stock solution was prepared by diluting 0.25 M ammonium iron(II) sulfate (reagent grade, Kanto Chemical) in 0.2 M HCl. A daily working solution of Fe(II) was made at concentration of 20 μM by diluting the stock solution with purified water. A stock solution of H_2_O_2_ was prepared at ~10 mM by diluting concentrated H_2_O_2_ (30.0–35.5% w/w, Kanto Chemical) in purified water. The concentration of H_2_O_2_ stock was determined by fluorescence measurement of the oxidized resorufin produced by a horseradish peroxidase catalyzed reaction [41]. A 0.5 mM luminol reagent was prepared monthly by dissolving luminol (5-amino-2,3-dihydro-1,4-phthalazinedione, Sigma Aldrich) in 1 M ammonia solution (reagent grade, Kanto Chemical). The pH of the luminol reagent was adjusted to 10.3 ± 0.1 with 5 M HCl. In the Fe(III) reduction experiment, a 0.5 M Fe(III) stock solution was prepared by diluting 1 g L^-1^ Fe(III) standard solution (iron(III) nitrate, reagent grade, Kanto Chemical) in 0.2 M HNO_3_. A 0.1 M ferrozine (Fz) solution was prepared by dissolving ferrozine (3-(2-pyridyl)-5,6-bis(4-phenylsulfonicacid)-1,2,4-triazine, Sigma Aldrich) in purified water followed by the pH adjustment to 7.0 ± 0.05 and 8.0 ± 0.05. Ferrozine has been employed for the measurement of photochemical and non-photochemical reduction of iron in natural waters by a number of previous studies in the last few decades [42–45]. A buffer solution containing 10 mM sodium chloride (reagent grade, Kanto Chemical) and 2 mM sodium bicarbonate (reagent grade, Kanto Chemical) was prepared at pH 7.0 ± 0.05 and 8.0 ± 0.05. The 2 mM sodium bicarbonate buffer has been often used for studies on the iron transformation kinetics in a simulated natural waters containing relatively high concentration of humic substances (e.g., order of milligram per litter) [46–47], as iron transformation kinetics including iron reduction and oxidation kinetics are dominantly controlled by organically complexed form under this condition. The solution was used as the reaction medium in the oxidation and reduction experiments in order to avoid significant changes of pH when chemicals were added to the medium.

### Fe(III) reduction experiment

Photochemical reduction experiment for Fe(III) complexed by standard HS was conducted by using a solar simulator (MS-35AAA, Ushio Lighting Edge Technologies, Japan, light intensity of 1 kW m^-2^) as a light source. Sample solutions were prepared in 1 cm path length spectrophotometer polystyrene cuvette containing the bicarbonate buffer and Fe(III)-HS complexes. First, the standard HS stock was mixed in bicarbonate buffer at final concentration of 200 mg L^-1^. Then, inorganic Fe(III) stock was added to mixtures of standard HS and buffer solution at a final concentration of 5 μM and the samples were stored overnight in the dark to achieve equilibrium. To avoid precipitation of Fe(III), sufficiently high HS and Fe concentration ratio was employed for most of HS samples given the published Fe(III) complexation capacities for standard HS at circumneutral pH [17]. For example, the published complexation capacities for standard HS used in this work ranged from 3.3 × 10^−8^ to 24 × 10^−8^ mol mg^-1^ at pH 6.5–8.0 [17], while Fe and HS concentration ratio used in the experiment (2.5 × 10^−8^ mol mg^-1^) was below these values. However, for the Pony Lake FA (0.21–0.58 × 10^−8^ mol mg^-1^), Inogashira Soil FA (1.9 × 10^−8^ mol mg^-1^), Biwa Lake FA (0.49–0.73 × 10^−8^ mol mg^-1^), the Fe and HS concentration ratio exceeded the binding capacity due to the limited availability of HS samples.

The photo-reduction experiment was initiated by addition of 1 mM Fz to the sample in the cuvette. During the light irradiation, Fz reacts with Fe(II) generated by photo-reduction to form Fe(II) complex (Fe(II)(Fz)_3_) that has a maximum absorbance at 562 nm. Thus, reduction of Fe(III) to Fe(II) under light irradiation was determined by measuring the time course of absorbance for Fe(II)Fz_3_ using a UV/vis spectrophotometer (U-2010, HITACHI, Japan). The absorbance at 562 nm was baseline-corrected at 750 nm. The Fe(II)(Fz)_3_ concentration was measured every 15–30 min for 2 h. The Fe(II)(Fz)_3_ concentration was calibrated with known concentrations of Fe(II) complexed by Fz. The molar absorption coefficient for Fe(II)(Fz)_3_ at 562 nm was determined to be 27,000 M^-1^ cm^-1^, which is consistent with literature value [48]. The thermal reduction of Fe(III) complexed by standard HS was also measured by using the procedure identical to that employed for the photochemical experiment, except that Fe(II)(Fz)_3_ formation was monitored in the dark condition. The concentration of Fe(II)(Fz)_3_ was measured every 1–1.5 h for 7 h. The change in pH of the reaction medium before and after the Fe(II) oxidation experiment was less than ± 0.05 unit.

The rate constants for the photo- and thermal reduction were obtained as follow:

$$\text{ln}\left(\frac{{\text{[Fe(III)]}}_{\text{0}}{-[\text{Fe}(\text{II})(\text{Fz})}_{\text{3}}{\text{]}}_{\text{t}}}{{\text{[Fe(III)]}}_{\text{0}}}\right){=\text{-k}}_{\text{red}}\text{t}$$ (1)

where k_red_ is the first order rate constant for Fe(III) reduction (s^-1^), [Fe(III)]_0_ is the initial concentration of Fe(III) present in sample (at the time when Fz was added to the sample), and [Fe(II)(Fz)_3_]_t_ is the concentration of Fe(II)(Fz)_3_ at given time (s). The k_red_ was obtained by fitting (Eq 1) to the experimental data by linear regression. In this study, rate constant obtained in the dark treatment was considered to be the thermal reduction rate constant (k_red_d_). The rate constant for the photochemical reduction (k_red_p_) was then calculated by subtracting the thermal reduction rate constant (i.e., k_red_d_) from the rate constant determined in the photochemical reduction experiment.

### Fe(II) oxidation experiment

Fe(II) oxidation rate constants were determined in the presence of various types of standard HS by measurement of the time course change of Fe(II) concentration using a flow injection analysis system equipped with a luminol chemiluminescence detector (FeLume, Waterville Analytical, USA) [49]. The Fe(II) oxidation experiment was initiated by standard addition of Fe(II) working solution at final concentrations of 10–50 nM to the air-saturated bicarbonate buffer solution containing 1 mg L^-1^ standard HS (pH 7.0–8.0). The Fe(II) oxidation experiment was performed with and without addition of 100 nM H_2_O_2_.

The sample and the luminol reagent were separately pumped using a peristaltic pump (RP-1, Rainin Instrument, CA) at a flow rate of 2.4 mL min^-1^ into the FeLume system and mixed in a flow cell situated in front of a photomultiplier tube (PMT, Hamamatsu) operating at -1200 V. The PMT signal was then recorded by WA control v91 software. The system was calibrated by recording the PMT signals of three standard addition of Fe(II). The signals were then corrected by subtracting the baseline signal (i.e., signal measured in the absence of Fe(II) addition). After the addition of Fe(II) into the reaction medium, there was a time lag (~50 s) to obtain stable signal. The initial signal was determined by extrapolating back to initial time (i.e., when Fe(II) was added) using linear regression analysis for the plot of log-transformed stable signal versus time. Then, by using the calibration line (log-linear plot between initial Fe(II) concentration and initial signal), the signal measured at arbitrary time was converted to the sample Fe(II) concentration. The system calibration was performed for each HS, because the signal intensity varied depending on the type of HS (due to the different degree of signal quenching effect of HS). The change in pH of the reaction medium before and after the Fe(II) oxidation experiment was less than ±0.05. Since the H_2_O_2_ concentration was negligibly low in the purified water used in this study, the oxidation by H_2_O_2_ was not considered in the experiment which omits the addition of H_2_O_2_.

The Fe(II) oxidation can be mediated by inorganic and organic oxidants present in natural waters [9, 50, 51]. However, dissolved oxygen is the main oxidant in air-saturated condition, whereas the H_2_O_2_ may be another important oxidant when H_2_O_2_ concentration is relatively high [9–11]. At nanomolar Fe concentrations, the contributions of reactive oxygen species (generated during the Fe(II) oxidation [i.e., Haber-Weiss mechanism] such as superoxide [O_2_^•-^] and hydroxyl radical [HO^•^]) to Fe(II) oxidation are recognized to be minor, as these highly reactive compounds can be scavenged by other competing reactions mediated by redox-reactive substances such as NOM [6,9,10]. Thus, oxidation rate of Fe(II) can be described as follow:

$$-\frac{\text{d[Fe(II)]}}{dt}{=\text{k}}_{\text{O2}}{\text{[Fe(II)][O}}_{\text{2}}{]+\text{k}}_{\text{H2O2}}{\text{[Fe(II)][H}}_{\text{2}}{\text{O}}_{\text{2}}\text{]}$$ (2)

where k_O2_ and k_H2O2_ (M^-1^ s^-1^) are the second-order rate constants for Fe(II) oxidation by O_2_ and H_2_O_2_, respectively. Under air-saturated condition at 25°C, dissolved oxygen concentration (~240 μM) is substantially higher than Fe(II) concentration. Therefore, Fe(II) oxidation by O_2_ (k_O2_) was assumed as pseudo-first-order reaction and rate constant (k*_O2_, s^-1^) was determined by linear regression analysis. Then, second-order rate constants were determined from k_O2_ = k*_O2_/[O_2_] for each HS. The rate constant for H_2_O_2_ mediated-oxidation (k_H2O2_) was then obtained for each HS by fitting the Eq 2 to the experimentally determined data (in the presence of H_2_O_2_) using Kintecus version 4. The rate constants were reported as the average of three standard additions of Fe(II).

### Determination of steady-state Fe(II) fraction

The steady-state Fe(II) fraction (Fe(II)]_ss_/[Fe]_T_) was estimated by using oxidation and reduction rate constants obtained in this study as follow:

$$\frac{{\text{[Fe(II)]}}_{\text{SS}}}{{\text{[Fe]}}_{\text{T}}}=\frac{{\text{k}}_{\text{red}\_\text{p}}{+\text{k}}_{\text{red}\_\text{d}}}{{\text{k}}_{\text{O2}}{\text{[O}}_{\text{2}}{]+\text{k}}_{\text{H2O2}}{\text{[H}}_{\text{2}}{\text{O}}_{\text{2}}\text{]}}$$ (3)

The computed steady-state Fe(II) fraction in the presence of simulated sunlight includes k_O2_, k_H2O2_, k_red_p_, and k_red_d_, whereas k_ox_, k_H2O2_, and k_red_d_ were used in the calculation for dark condition.

### Statistical analysis

To examine the relationships between Fe redox rate constants and chemical properties of standard HS, the Spearman's rank correlation coefficients (non-normally distributed data) and the Pearson's correlation coefficient (normally distributed data) were applied according to the Kolmogorov-Smirnov test. Linear regression analysis for Fe(II) oxidation and Fe(III) reduction was performed to the data in the plot of signal versus time. Statistical analysis was performed using statistical software R.

## Results and discussion

### Redox rate constants

**Fig 1:**
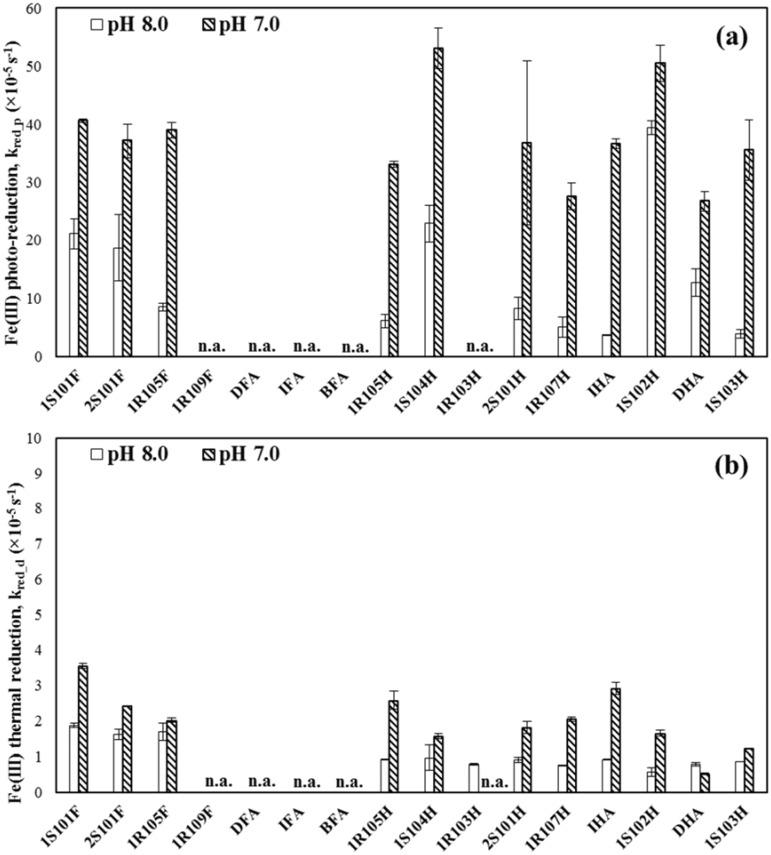
Fe(III) reduction rate constants (a) in the presence of simulated sunlight and (b) under dark conditions for various types of standard HS at pH 7.0 and 8.0. Error bars indicate standard deviation from triplicate measurements. “n.a.” indicates no data available.

The first-order rate constants for Fe(III) photo-reduction (k_red_p_) varied by a factor of 10 from 3.8 × 10^−5^ to 3.9 × 10^−4^ s^-1^ at pH 8.0 and by a factor of 2.0 from 2.7 × 10^−4^ s^-1^ to 5.3 × 10^−4^ s^-1^ at pH 7.0, depending on the type of standard HS (Table 1 and Fig 1a). The Fe(III) photo-reduction rate constants at pH 7.0 in average were determined to be greater than or comparable to those for the k_red_p_ at pH 8.0. In addition, the Fe(III) thermal reduction rate constants (k_red_d_) also showed the similar trend except for DHA: for example, the measured rate constants ranged from 5.3 × 10^−6^ to 3.5 × 10^−5^ s^-1^ at pH 7.0 (i.e., 6.6-fold change depending on the type of standard HS), which were higher than or comparable to those at pH 8.0 (ranging from 5.6 × 10^−6^ to 1.9 × 10^−5^ s^-1^: i.e., 3.4-fold change) (Fig 1b). On average, the Fe(III) photo-reduction rates were greater than thermal reduction rates by 13-fold at pH 8.0 and 19-fold at pH 7.0.

**Table 1:** Iron (Fe) redox rate constants and steady-state Fe(II) fraction ([Fe(II)]_ss_/[Fe]_T_) for each standard HS samples.

Code of SHS	k_O2_ (M^-1^ s^-1^)		k_H2O2_ (× 10^3^ M^-1^ s^-1^)		k_red_d_ (× 10^−6^ s^-1^)		k_red_p_ (× 10^−5^ s^-1^)		[Fe(II)]_ss_/[Fe]_T_ (%), dark		[Fe(II)]_ss_/[Fe]_T_, light	
	pH 8.0	pH 7.0	pH 8.0	pH 7.0	pH 8.0	pH 7.0	pH 8.0	pH 7.0	pH 8.0	pH 7.0	pH 8.0	pH 7.0
2S101H	31.4 (1.6)	6.13 (1.73)	41.0 (11.0)	6.51 (0.93)	9.02 (0.68)	18.1 (1.8)	8.20 (1.97)	36.8 (14.1)	0.0941 (0.0793–0.112)	0.996 (0.715–1.47)	0.942 (0.668–1.27)	17.7 (9.72–28.3)
1S102H	17.4 (1.9)	5.57 (0.28)	7.11 (5.08)	1.18 (0.62)	5.63 (1.26)	16.5 (0.9)	39.4 (1.2)	50.5 (3.1)	0.124 (0.0830–0.180)	1.17 (1.03–1.33)	8.09 (6.85–9.74)	27.2 (24.7–29.9)
1S103H	22.4 (1.9)	8.05 (1.12)	19.7 (5.8)	3.52 (0.32)	8.60 (0.27)	12.3 (0.0)	3.96 (0.73)	35.6 (5.2)	0.135 (0.117–0.158)	0.579 (0.509–0.670)	0.753 (0.568–0.986)	14.9 (11.7–18.7)
1S104H	22.4 (1.9)	6.62 (1.23)	8.62 (6.24)	2.94 (0.36)	9.68 (3.60)	15.7 (0.7)	22.9 (3.2)	53.0 (3.5)	0.166 (0.0922–0.262)	0.899 (0.728–1.14)	3.94 (2.99–5.15)	23.9 (20.0–29.0)
1R103H	27.2 (2.4)	7.55 (1.90)	19.7 (5.8)	3.09 (0.20)	7.83 (0.18)	(n.m.)	(n.m.)	(n.m.)	0.104 (0.0912–0.120)	(n.a.)	(n.a.)	(n.a.)
1R105H	29.5 (5.2)	7.20 (2.22)	20.5 (5.6)	5.27 (0.74)	9.16 (0.07)	25.8 (2.5)	6.07 (1.08)	33.1 (0.5)	0.113 (0.0940–0.140)	1.28 (0.901–1.95)	0.853 (0.607–1.21)	15.2 (12.0–20.4)
1R107H	19.9 (4.7)	10.6 (2.7)	49.8 (2.6)	4.01 (0.80)	7.50 (0.10)	20.5 (0.6)	5.03 (1.67)	27.6 (2.3)	0.103 (0.0865–0.126)	0.743 (0.575–1.02)	0.789 (0.478–1.23)	9.77 (7.37–13.5)
DHA	45.8 (2.2)	10.5 (2.3)	22.2 (2.5)	4.42 (0.77)	7.87 (0.43)	5.29 (0.15)	12.7 (2.4)	26.8 (1.7)	0.0650 (0.0583–0.0724)	0.192 (0.154–0.250)	1.10 (0.860–1.37)	9.02 (7.12–11.8)
IHA	24.5 (4.5)	9.41 (0.85)	21.8 (2.6)	0.589 (0.524)	9.20 (0.88)	29.2 (1.7)	3.75 (0.08)	36.7 (0.8)	0.132 (0.101–0.174)	1.26 (1.08–1.48)	0.665 (0.546–0.832)	14.8 (13.3–16.5)
1S101F	26.0 (2.0)	5.28 (3.01)	46.2 (14.5)	5.61 (1.08)	18.7 (0.6)	35.5 (0.7)	21.1 (2.6)	40.6 (0.3)	0.218 (0.185–0.261)	2.24 (1.47–4.49)	2.62 (2.04–3.38)	22.2 (15.8–36.6)
2S101F	49.8 (3.1)	4.30 (1.57)	60.8 (11.1)	6.23 (1.13)	16.2 (1.5)	24.3 (0.1)	18.7 (5.7)	37.2 (2.9)	0.108 (0.0905–0.129)	1.78 (1.35–2.62)	1.33 (0.880–1.87)	22.8 (17.1–31.9)
1R105F	37.9 (5.2)	6.13 (2.16)	26.7 (1.9)	5.16 (0.68)	16.9 (2.5)	20.2 (0.6)	8.46 (0.69)	39.0 (1.3)	0.162 (0.122–0.213)	1.16 (0.853–1.74)	0.964 (0.775–1.21)	19.2 (14.8–26.5)
1R109F	37.7 (2.1)	2.16 (1.94)	87.3 (15.7)	7.51 (1.79)	(n.a.)	(n.a.)	(n.a.)	(n.a.)	(n.a.)	(n.a.)	(n.a.)	(n.a.)
DFA	24.3 (1.3)	4.79 (1.31)	47.4 (13.2)	6.59 (0.87)	(n.m.)	(n.m.)	(n.m.)	(n.m.)	(n.a.)	(n.a.)	(n.a.)	(n.a.)
IFA	31.5 (1.1)	1.81 (1.73)	40.8 (9.7)	7.17 (0.77)	(n.a.)	(n.a.)	(n.a.)	(n.a.)	(n.a.)	(n.a.)	(n.a.)	(n.a.)
BFA	33.8 (1.5)	1.54 (2.08)	103 (7)	7.45 (1.63)	(n.a.)	(n.a.)	(n.a.)	(n.a.)	(n.a.)	(n.a.)	(n.a.)	(n.a.)
Inorganic Fe	8.8 ^b^	0.38 ^b^	37.2 ^b^	4.79 ^b^	(n.a.)	(n.a.)	(n.a.)	(n.a.)	(n.a.)	(n.a.)	(n.a.)	(n.a.)

(n.a.): not applicable; (n.m.): not measured.

^*a*^ The values in parenthesis represent a standard deviation (SD) and the range estimated by taking the upper and lower limits for the SD of redox rate constants into account for the redox rate constants and [Fe(II)]ss/[Fe]_T_, respectively.

^*b*^ Inorganic Fe(II) oxidation rate in the 2 mM NaHCO_3_ and 0.1 M NaCl from Pham and Waite (2008) [5].

The Fe(III) reduction rate constants determined in this study were reasonably comparable to the reported values for Fe(III)SRFA complex at pH 8.0 in previous study (e.g., 1.3 × 10^−5^ s^-1^ for the thermal reduction and 1.7 × 10^−4^ s^-1^ for the photochemical reduction in the presence of solar simulator, Fujii et al. [52]). The photo-reduction rates for Fe(III)-HS complexes were also comparable to the literature values for EDTA as a Fe-binding ligand (e.g., 1.2 × 10^−4^ s^-1^ under the solar simulator at pH 8.0) [52]. It should be noted that inorganic Fe(III) is also reduced via direct photolysis. For example, Fe^III^OH^2+^ is known to be photochemically reactive forming Fe^2+^ and OH• radical [31]. At circumneutral pH (e.g., pH 7–8), however, Fe^III^(OH)_2_^+^, Fe^III^(OH)_3_^0^ and Fe^III^(OH)_4_^-^ account for a majority of total dissolved inorganic Fe(III) and Fe^III^OH^2+^ is a minor species [12]. Thus, it is unlikely that direct photo-reduction of inorganic Fe(III) is important under the conditions examined in this study [53].

**Fig 2:**
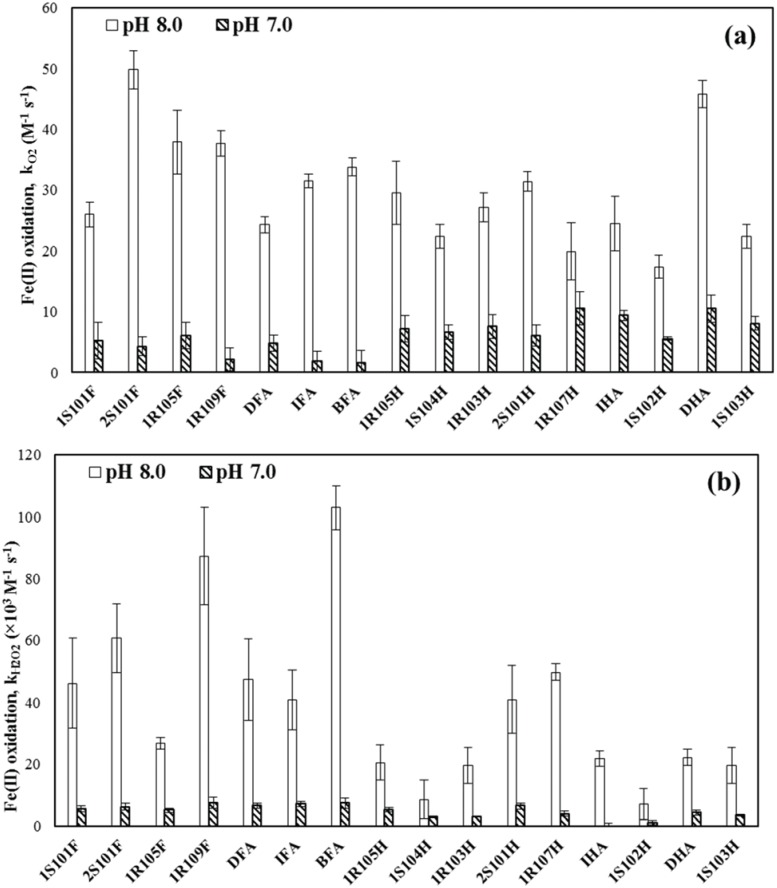
Fe(II) oxidation rate constants by (a) dissolved oxygen and (b) hydrogen peroxide for various standard HS at pH 7.0 and 8.0. Error bars represent standard deviation (n = 3).

The Fe(II) oxidation rate constants (k_O2_) by O_2_ were also depending on the type of HS and determined to be 17.4–49.8 M^-1^ s^-1^ at pH 8.0 (i.e., 2.9-fold change) and 1.54–10.6 M^-1^ s^-1^ at pH 7.0 (i.e., 6.9-fold change) (Fig 2a). The results indicated that k_O2_ at pH 8.0 were overall greater than those for pH 7.0. At pH 8.0, the oxidation rates were comparable to those previously found for the Fe complexes with various types of HS in seawater (5.6–52 M^-1^ s^-1^ [8]). Consistent with previous literature, Fe(II) oxidation rate constants by O_2_ increased with increasing pH [9,10,18,19,54,55]. Our oxidation rates for Fe complexed by HS were substantially higher than the reported value for inorganic Fe(II) oxidation by O_2_ for 0.1 M NaCl at pH 8.0 (8.8 M^-1^ s^-1^ [5]), indicating that the complexation by HS accelerates Fe(II) oxidation.

The Fe(II) oxidation rate constants by H_2_O_2_ (k_H2O2_) at pH 8.0 ranged from 7.1 × 10^3^ to 1.0 × 10^5^ M^-1^ s^-1^ (i.e., 15-fold change) and from 5.9 × 10^2^ to 7.5 × 10^3^ M^-1^ s^-1^ (i.e., 13-fold change) depending on the type of HS. The oxidation experiment indicated that effect of HS type on Fe(II) oxidation was larger for the H_2_O_2_-mediated oxidation compared to the O_2_-mediated oxidation. Overall, the k_H2O2_ at pH 8.0 were higher than the k_H2O2_ at pH 7.0 (Fig 2b). The measured oxidation rates (k_H2O2_) at pH 8.0 were slightly lower or comparable to the previously reported values for the H_2_O_2_-mediated oxidation of dissolved inorganic Fe(II) by Rose and Waite [6] in seawater at pH 8.1 (3.1 × 10^4^ M^-1^ s^-1^) and also by Miller et al. [47] for freshwater at pH 8.4 (~10^5^ M^-1^ s^-1^). The latter study also reported that the complexation of Fe(II) by SRFA retards the H_2_O_2_-mediated oxidation (e.g., ~10^3^ M^-1^ s^-1^ at pH 8.4).

### Steady-state Fe(II) fraction

**Fig 3:**
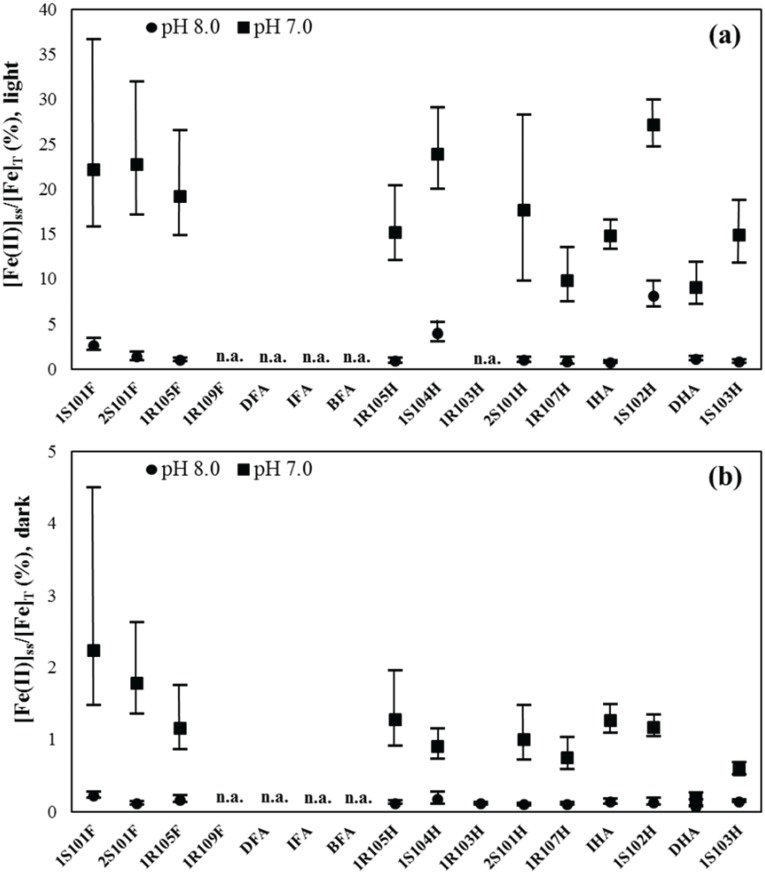
Computed steady-state Fe(II) fractions (a) in the presence of simulated sunlight and (b) under dark conditions for standard HS at pH 7.0 and 8.0. The upper and lower whiskers indicate maximum and minimum range values, respectively (see Table 1 for detailed values). “n.a.” indicates no data available.

The computed steady-state Fe(II) fraction in the presence of simulated sunlight ranged from 0.66–8.1% at pH 8.0 and 9.0–27% at pH 7.0 (Fig 3a), indicating that the Fe(II) fraction varies by 12-fold at pH 8.0 and 3-fold at pH 7.0 in the presence of various HS examined. In average, the steady-state Fe(II) fraction in the presence of simulated sunlight at pH 7.0 was higher than that for pH 8.0 by a factor of 8.9-fold. Under the dark condition, Fe(II) fraction varied from 0.06–0.22% at pH 8.0 (3.7-fold change) and 0.58–2.2% at pH 7.0 (3.8-fold change), depending on HS type (Fig 3b). Similar to the light system, the steady-state Fe(II) fraction at pH 7.0 was in average higher than the Fe(II) fraction at pH 8.0. Compared to the dark condition, the steady-state Fe(II) fraction in the presence of simulated sunlight largely increased by 20-fold for pH 7.0 and 16-fold for pH 8.0. The higher steady-state Fe(II) fractions in both dark and simulated sunlight conditions found at pH 7.0 are consistent with faster photochemical and thermal reduction and slower oxidation rates and vice versa for pH 8.0 (see also Figs 1a and 2a).

**Fig 4:**
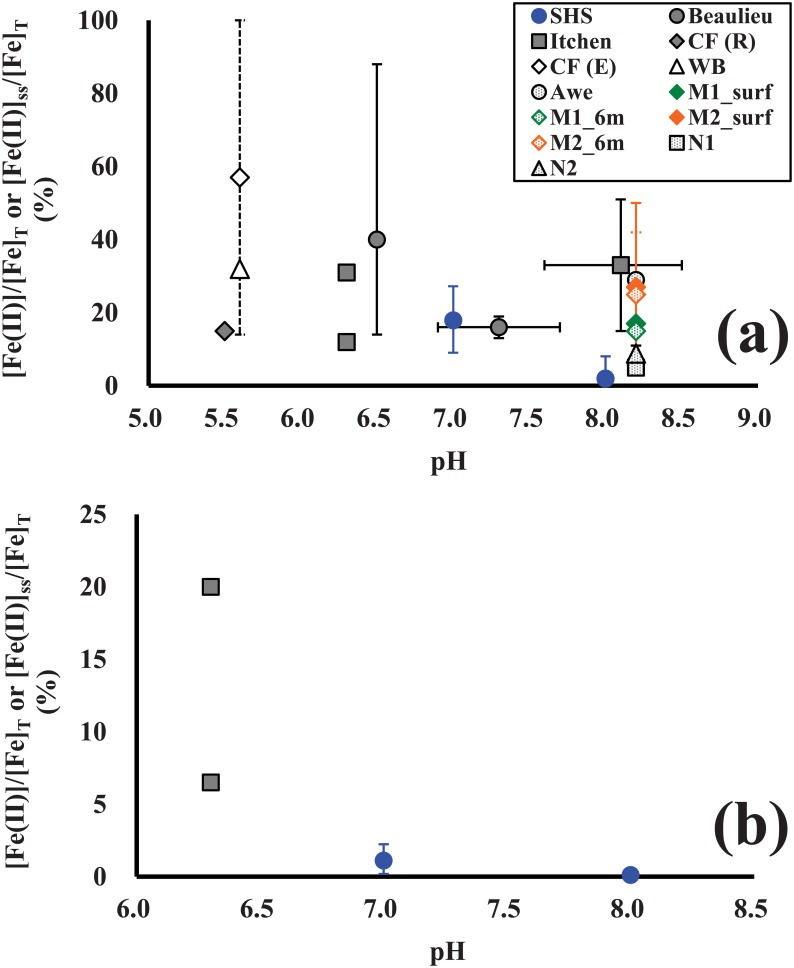
Fe(II) fractions ([Fe(II)]/[Fe]_T_, or [Fe(II)]_ss_/[Fe]_T_) in fresh and estuarine waters, as well as solutions of standard HS (SHS) and size-fractionated NOM, plotted as a function of pH: (a) the Fe(II) fractions during daytime or in the presence of simulated sunlight and (b) the Fe(II) fractions during night-time or in the dark condition. Source data and abbreviation for each site or sample are summarized in Table 2. A data point for the site or sample with *n* ≥ 2 represents the mean value (represented by symbols) and their minimum and maximum values (represented by the upper and lower whiskers, respectively, except for N1 and N2 with whiskers indicating the standard deviation). Data points for M1 and M2 indicate the mean values for high-molecular-weight and low-molecular-weight NOM fractions.

The computed steady-state Fe(II) fractions in this study (0.06–27%) were reasonably consistent with the reported values on the measurement of dissolved Fe(II) concentration relative to that of total dissolved Fe (i.e., the Fe(II) fraction: [Fe(II)]/[Fe]_T_) for river, lake and estuarine waters and NOM extracts (5–100%) (Table 2 and Fig 4). The Fe(II) fraction observed in the natural waters and NOM extracts in previous studies appeared to increase as pH decreases in the presence and absence of sunlight, which is in accordance with our results for the standard HS solutions (Fig 4**)**. In the presence of light, however, [Fe(II)]_ss_/[Fe]_T_ for the standard HS solution at pH 8.0 (0.66–8.1%) was a little lower than [Fe(II)]/[Fe]_T_ in the other natural waters or NOM solutions (e.g., River Itchen, Lake Murtensee) in a similar pH range (3–51%) (Fig 4). One of the plausible explanations for this difference is that dissolved components from the anthropogenic inputs (e.g., sewage effluents, agricultural drainage) affect the Fe redox kinetic in the natural waters and NOM samples in previous studies. For example, Hopwood et al. [56] indicated that Fe(II) and other trace metals (e.g., copper and zinc) form relatively stable dissolved sulfide complexes at up to several hundred nanomolar concentrations in river, estuarine and coastal waters affected by wastewater discharges [57–59]. Indeed, the sewage effluent was estimated to account for 10% of freshwater waters in the River Itchen [56]. In addition, according to a study by Meunier et al. [35], a higher [Fe(II)]/[Fe]_T_ was observed in the lake water (M2) close to the city (Murten), as compared to that at middle of the lake (M1) (Table 2). Investigation on dissolved sulfides and other components forming stable Fe(II) complexes and its influence in the Fe redox speciation may warrant a future study to examine the Fe(II) formation and bioavailability in the anthropogenically-impacted aquatic systems. Furthermore, water temperature potentially affects the Fe redox kinetics [10,54,55]: therefore, water temperature should be also considered for the formation of Fe(II) in natural and anthropogenically-impacted waters. In addition to these factors, the contributions of photochemically generated oxidants as well as the higher degree of re-complexation of generated Fe(II) by NOM due to the relatively higher HS concentration (and/or Fe-binding affinity), which were not taken into account in the calculation, may be other plausible candidates for the lower Fe(II) fraction in this study.

**Table 2:** Summary of ferrous iron (Fe[II]) fraction (concentration of dissolved Fe[II] relative to that of total dissolved iron; i.e., [Fe(II)]/[Fe]_T_) found in natural waters and solutions of natural organic matter (NOM).

Site or sample	Water type	Station Code	Abbreviation in Fig 4	*N*	pH	[Fe(II)] (nM)^a^	[Fe]_T_ (nM)^b^	[Fe(II)]/[Fe]_T_ (%)	Remarks	Ref.
**(i) Daytime (or in the presence of simulated sunlight)**										
Solution of standard humic substances (SHS)	Estuarine water (assumed)	-	SHS	11	8.0	-	-	2.00 (0.665–8.09)^c^^,^ ^d^		This study
				11	7.0	-	-	17.9 (9.02–27.2)^c^^,^ ^d^		
River Beaulieu, UK	River water	-	Beaulieu	9	6.5	2,900–14,000	11,400–20,500	40 (14–88)^c^	Samples were collected at 11:00 on 28 Jan. 2013.	[60]
		-		12	6.9–7.7	180–3,600	1,400–19,000	13–19^e^		[56]
Beaulieu estuary, UK	Estuarine water	-	-	2	(n.a.)	(n.a.)	(n.a.)	30–31	Salinity (*S*) = 0.	[60]
River Itchen, UK	River water	-	Itchen	5	7.6–8.5	12–230	79–450	15–51^e^		[56]
		-		1	6.3	44	360	12	Sample was collected at 11:20 on 24 Oct. 2012.	
		-		1	6.3	55	180	31	Sample was collected at 15:20 on 24 Oct. 2012.	
Itchen Estuary, UK	Estuarine water	-	-	1	(n.a.)	238	623	38	*S* = 0.29.	[61]
Cape Fear, NC	River water	-	CF (R)	1	5.5	770	5,200	15		[56]
	Estuarine water	-	CF (E)	12	5.6^f^	46–700	38–5,000	14–100^e^		
Winyah Bay, SC	Estuarine water	-	WB	4	5.6^f^	30–1,900	92–6,200	31–33^e^		[56]
River Awe, UK	River water	-	Awe	1	8.2	170	580	29		[56]
Murtensee, Switzerland (NOM extract)	Lake water (surface)	M1	M1_surf	1	8.2^g^	-	-	4–29^d^^,^ ^h^	M1 is located in the middle of the lake.	[35]
	Lake water (6 m in depth)		M1_6m	1	8.2^g^	-	-	5–25^d^^,^ ^h^		
	Lake water (surface)	M2	M2_surf	1	8.2^g^	-	-	3–50^d^^,^ ^h^	M2 is located offshore of the city of Murten.	
	Lake water (6 m in depth)		M2_6m	1	8.2^g^	-	-	7–42^d^^,^ ^h^		
River Scheldt, Belgium (NOM extract)	River water	N1	N1	1	8.2^g^	-	-	5.0 (0.6)^d^^,^ ^i^		[35]
River Waal, Netherlands (NOM extract)	River water	N2	N2	1	8.2^g^	-	-	8.8 (2.2)^d^^,^ ^i^		[35]
**(ii) Night-time (or in the dark)**										
SHS solution	Estuarine water (assumed)	-	SHS	12	8.0	-	-	0.127 (0.0650–0.218)^c^^,^ ^d^		This study
				11	7.0	-	-	1.12 (0.192–2.24)^c^^,^ ^d^		
River Itchen, UK	River water	-	Itchen	1	6.3	81	400	20	Sample was collected at 7:20 on 24 Oct. 2012.	[56]
		-		1	6.3	26	400	6.5	Sample was collected at 18:30 on 24 Oct. 2012.	

(n.a.): no data or not applicable.

^*a*^ Concentration of dissolved Fe(II).

^*b*^ Concentration of total dissolved iron.

^*c*^ Mean value followed by range in parenthesis.

^*d*^ Value at steady-state.

^*e*^ Minimum or maximum values are calculated either as minimum [Fe(II)] relative to minimum [Fe]_T_ or maximum [Fe(II)] relative to maximum [Fe]_T_.

^*f*^ Value for freshwater.

^*g*^ pH under which steady-state Fe(II) fraction was examined.

^*h*^ Mean value of 2–3 measurements. Minimum and maximum values refer to the value for high-molecular-weight (HMW; > 1 kDa) and low-molecular-weight (LMW; < 1 kDa) DOM fractions, respectively.

^*i*^ Mean value of 2–3 measurements followed by mean deviation in parenthesis for HMW DOM fraction.

### Effect of HS molecular composition on Fe redox rate constants and steady-state Fe(II) fraction

**Fig 5:**
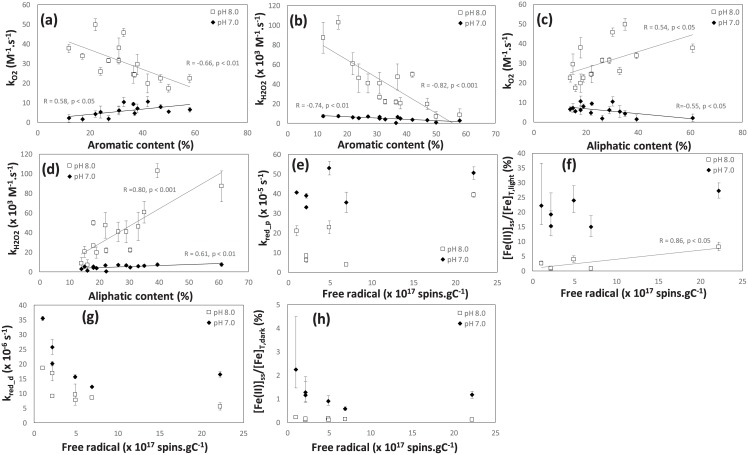
Relationships between major parameters for HS chemical properties (e.g., aromatic and free radical contents) and redox rate constants or steady-state Fe(II) fractions determined in this study: (a) aromatic (in x-axis) vs. k_O2_ (in y-axis), (b) aromatic vs. k_H2O2_, (c) aliphatic vs. k_O2_, (d) aliphatic vs. k_H2O2_, (e) free radical vs. k_red_p_, (f) free radical vs. [Fe(II)]_ss_/[Fe]_T, light_, (g) free radical vs. k_red_d_, and (h) free radical vs. [Fe(II)]_ss_/[Fe]_T, dark_. In each figure panel, the data at pH 7.0 and 8.0 were presented. Mean values were indicated by symbols. Error bars for the redox rate constants represent standard deviation. Minimum and maximum values for the steady-state Fe(II) fractions (i.e., [Fe(II)]_ss_/[Fe]_T,dark_ and [Fe(II)]_ss_/[Fe]_T,light_) were represented by upper and lower whiskers, respectively. If significant correlations were observed for the parameters, liner regression lines were inserted with correlation coefficient and p-value.

To investigate the variation of redox rate constants and resultant Fe(II) formation in a range of HS, correlations of Fe redox rate constants and steady-state Fe(II) fractions with molecular compositions of HS were examined (Fig 5, S1 Fig and S2 Table). Statistically significant negative correlations (p < 0.05) were found between aromatic carbon content (and aromaticity) of HS and oxidation rates for k_O2_ (r = -0.66 for pH 8.0) and k_H2O2_ (r = -0.82 for pH 8.0 and r = 0.74 for pH 7.0) (except for k_O2_ with r = 0.58 at pH 7.0) (Fig 5a and 5b). Similarly, the hydrogen to carbon ratio (H/C ratio) showed significant positive correlations (p < 0.05) with k_O2_ (e.g., r = 0.65 for pH 8.0) and k_H2O2_ (e.g., r = 0.62 for pH 7.0 and 0.79 for pH 8.0), and no significant correlations were found for k_O2_ at pH 7.0. The relationships between aliphatic carbon content and oxidation rate constants were similar to those for aromatic carbon content but in an inverse manner (Fig 5c and 5d). These results generally suggest that Fe(II) oxidation by O_2_ and H_2_O_2_ are accelerated via the Fe(II) complexation by HS with a high aliphatic content. The results are consistent with a finding in the previous study [18], where significant negative correlation was observed between the specific UV absorbance (SUVA_254_, an index of DOM aromaticity) and Fe(II) oxidation rate constant (pH 8.0) in the Sagami River waters, Japan. Interestingly, in some cases, SUVA_254_ was found to have higher correlation coefficients with oxidation rate constants compared to those for the aromaticity and aromatic content in our correlation analyses (S2 Table). Since it is recognized that the Fe(II) oxidation is affected by the relative binding affinity of NOM to Fe(II) and Fe(III) (according to the linear free energy relation theory) [20,50], the binding affinity for Fe(III) relative to that for Fe(II) might be higher for HS with a higher aliphaticity. In addition, previous studies indicated that aliphatic structures of NOM provide strong metal-binding ligands through multidentate coordination with trace metals [62–64]. Nonetheless, the mechanism behind the inverse relation for k_O2_ and aliphatic content at different pH (i.e., negative and positive relations at pH 7 and pH 8, respectively: e.g., Fig 5c and S2 Table) remains unclear, though it may be associated with the fact that major Fe(II) species involved in Fe(II) oxidation vary depending on pH, as discussed in the Supporting Information (S1 File).

The reduction rate constant and steady-state Fe(II) fractions in the presence of simulated sunlight had relatively strong positive relations with free radical content (r = 0.74 and r = 0.54 for reduction rate at pH 8.0 and pH 7.0 respectively: r = 0.86 and r = 0.60 for Fe(II) fractions at pH 8.0 and pH 7.0, respectively: S2 Table, Fig 5e and 5f), though significan correlation (p < 0.05) was only observed for the Fe(II) fractions at a higher pH (i.e., 8.0) possibly due to the small number of samples (n = 6–7) and/or inner filter effect in the presence of the relatively high concenraiton of HS, as discussed in the Supporting Information (S2 File). Nevertheless, the result is generally consistent with the previous finding that photochemically generated Fe(II) is relatively stable in the presence of salicylic acid, benzoic acid or 2,4-dichlorophenol owing to the reductive properties of the quinone-like intermediates [33,65]. Previous studies indicated quinone-like moieties are abundant as redox-active components in HS and semiquinone is characterized by free radicals [66,67]. In addition, the reduced state of quinones such as radical semiquinone and hydroquinone facilitate the reduction of Fe(III) generating Fe(II) and benzoquinone [68]. Therefore, one of the plausible reasons for the positive correlation between free radical content and photochemical reduction rate are due to the reductive property of quinone-like moieties in HS. The correlation of photochemical Fe(II) formation and free radical content is consistent with the relatively strong negative correlation between Fe(II) oxidation and free radical content in HS (r = -0.70 for O_2_ at pH 8.0: r = -0.63 and r = -0.87 for H_2_O_2_ at pH 8.0 and pH 7.0, respectively, S2 Table and S1 Fig). Nonetheless, the thermal reduction rate and steady-state Fe(II) fraction in the dark did not have positive correlations with the free radical content (Fig 5g and 5h) (note that free radical in HS does not include hydroquinone, another important quinone-like reductant). Although detailed mechanism behind these correlations will be warranted in future study, our correlation analysis implies that the net photochemical Fe(II) generation rate, which can be determined by the balance of photo-driven Fe(II) formation and concurrently occurring re-oxidation of Fe(II), is likely associated with the reductive properties of free radicals in HS.

In the LMCT reaction, dissolved Fe(III) species complexed by carboxylic acid ligands are reduced to Fe(II) and the carboxylic ligands are concurrently oxidized to carbon dioxide (CO_2_; decarboxylation) [31]. Iron carboxylate complexes have different photo-reactivities depending on the ligand types such as oxalate, malonate and monocarboxylates and their roles in secondary radical reactions [33]. For example, the Fe(III) complex with oxalate absorbs the near-UV light and undergoes efficient LMCT direct photolysis due to the formation of radical species (e.g., CO_2_^•-^) with a reductive ability [33]. The Fe complex with malonate Fe(III) also undergoes efficient direct photolysis, though the radical species formed by the photolysis of Fe(III)-malonate have an oxidative ability. Monocarboxylates have weak interactions with Fe(III) and are not involved in the direct photolysis [33]. However, in this study, no strong relations (r = 0.10–0.25, S2 Table) were observed between photochemical Fe(III) reduction and content of carboxyl functional group, suggesting that carboxyl content in HS does not represent the photo-reactivities of Fe(III) complexes. In contrast, thermal reduction rates have relatively strong positive correlations with carboxyl content (r = 0.82, p < 0.01 for pH 8.0 and r = 0.55 for pH 7.0, S2 Table). A previous study found that bio-reduction of Fe(III) minerals in soil was accelerated in the presence of HS with a higher carboxyl content presumably due to the complexation of Fe(II) by the carboxyl moieties [29], indicating that thermal Fe(III) reduction in natural waters could be enhanced by NOM rich in carboxyl functional groups. Our study also indicated that carboxyl is likely the important qualitative factors that affect the thermal reduction of Fe(III) complexed by HS.

The photo-decarboxylation process of NOM in the colored river waters of Georgia has been suggested to involve a simultaneous regeneration of carboxyl groups via oxidative cleavage of aromatic rings [31,69], resulting in the loss of aromatic moieties. In addition, the previous study indicated that, when aliphatic compounds (e.g., oxalic acid, citric acid and formaldehyde) were present, the photo-produced intermediate radicals are apt to generate active oxidizing species by the reaction with O_2_ [33,65]. These results are consistent with the negative relations of photochemical Fe(III) reduction and Fe(II) fraction with non-aromatic content (e.g, total carbohydrate with r = -0.39 − -0.63 for photochemical Fe(III) reduction and r = -0.40 − -0.51 for Fe(II) fraction, S2 Table), though significant correlation (p < 0.05) was only observed for total carbohydrate with photochemical Fe(III) reduction at pH 7.0. Overall, our correlation analysis indicated that variation of Fe redox rate constant and resultant Fe(II) formation is associated with some specific parameters for HS molecular composition.

## Conclusions

In this study, Fe redox rate constants and steady-state Fe(II) fractions were determined at circumneutral pH (pH 7–8) for various standard HS from different origins. Depending on the type of HS, the first-order rate constants for photochemical reduction substantially varied by up to 10-fold. The thermal reduction rates also varied by up to 7-fold. The first-order rate constants for photochemical and thermal Fe(III) reduction were pH-dependent and the rates at lower pH (i.e., pH 7) in average were determined to be higher than the reduction rates at higher pH (i.e., pH 8). The average Fe(III) photo-reduction rates were higher than the thermal reduction rates by 13–19 folds.

The Fe(II) oxidation rate constants were also depending on the type of HS and the degree of the effect of HS type on Fe(II) oxidation was larger for the H_2_O_2_-mediated oxidation compared to the O_2_-mediated oxidation (e.g., 15-fold and 3-fold changes at pH 8 for the H_2_O_2_- and O_2_-mediated oxidation, respectively). The Fe(II) oxidation rate constants were also pH-dependent and the oxidation rates at pH 8 were greater than those for pH 7. The computed steady-state Fe(II) fraction under the simulated sunlight indicated that the Fe(II) fraction varies by up to 12-fold. Under the dark condition, Fe(II) fraction varied by 4-fold change (at respective pH), depending on HS type. Due to the higher reduction rate and lower oxidation rate at lower pH, the computed steady-state Fe(II) fraction in the presence of simulated sunlight at the lower pH was higher than that for the higher pH by a factor of ~9-fold in average. The calculated Fe(II) fractions were reasonably consistent with the previously measured Fe(II) fraction for natural water samples.

The correlation analysis indicated that Fe(II) oxidation by O_2_ and H_2_O_2_ is facilitated due to the increasing degree of complexation of Fe(II) by HS with a high aliphatic content. The reduction rate constant and steady-state Fe(II) fractions in the presence of sunlight have positive relations with free radical content, possibly due to the reductive property of quinone-like moieties in HS. Although the Fe(III) coordination by carboxyl functional groups is important in LMCT process, no strong relations were observed between photochemical Fe(III) reduction and content of carboxyl functional group in this study, suggesting that carboxyl content does not represent the photo-reactivity of Fe(III) complexes. Our results indicated that Fe redox kinetics and resultant Fe(II) formation in the presence of HS are substantially influenced by molecular composition of HS.

## Supporting information

S1 Fig Relationships among the major parameters for HS chemical properties (e.g., aromatic and free radical contents), redox rate constants and steady-state Fe(II) fractions determined in this study. The correlation analysis was conducted for the purpose of visual inspection of the correlation among the parameters by using the statistical software R. In this survey, only the Pearson's correlation coefficients were shown.
(EPS)

S2 Fig UV-VIS absorbance spectrum for 250 mg/L SRFA (in the buffer solution) from 300 nm to 700 nm. The absorbance was baseline corrected by using the absorbance spectrum for the buffer solution.
(EPS)

S1 File Inverse relation for k_O2_ at different pH. (DOCX)

S2 File Inner filter effect in the photochemical experiment. (DOCX)

S1 Table Molecular properties of standard humic substances used in this study. (DOCX)

S2 Table Correlation coefficients (*r*) of iron (Fe) redox rate constants and steady-state Fe(II) fraction obtained in the presence of standard HS with the molecular parameters of HS. (DOCX)

S3 Table The light absorbed by SRFA in the photochemical experiment. (DOCX)
