# Supplementary material for: Variation of iron redox kinetics and its relation with molecular composition of standard humic substances at circumneutral pH
Source: PLoS One. 2017 Apr 28;12(4):e0176484. doi: 10.1371/journal.pone.0176484 (PMC5409151; doi:10.1371/journal.pone.0176484)
Supplement: S1 File — (DOCX) [file pone.0176484.s003.docx]

**S1 File. Inverse relation for k_O2_ at different pH**

Regarding the inverse relation for k_O2_ at different pH (i.e., negative and positive relations at pH 7 and pH 8, respectively), the mechanism behind this relation remains unclear, though it may be associated with the fact that major Fe(II) species involved in Fe(II) oxidation vary depending on pH. For example, in case of inorganic Fe(II) oxidation by O_2_, Fe^2+^ and FeCO_3_ are major species (~60%) at pH 7 in 0.1 M NaCl buffered by 2 mM NaHCO_3_ (Pham and Waite, 2008). At pH 8, incontrast, ~80% of overall Fe(II) oxidation rate is accounted for by the FeCO_3_(OH)^-^ and Fe(CO_3_)_2_^2-^. (see Fig 5 in Pham and Waite, 2008). While it is likely that iron oxidation kinetics are dominantly controlled by organically complexed forms in a simulated natural waters containing relatively high concentration of humic substances (as noted in the main text), the pH-dependent changes of Fe(II) oxidation observed in this study suggest that Fe(II) oxidation in the presence of HS is still affected by degree of the coordination of inorganic ligand(s) (e.g., OH^-^, CO_3_^2-^) to metal center in the Fe-HS complex. Therefore, one of the plausible explanations for the inverse relation observed may be due to the reversed response of oxidation of major Fe(II) species (K_O2_) to the HS quality changes (i.e., aliphatic content in this case) between pH 7 and 8. While Fe(II) oxidation rate constants were measured only at pH 7 and 8 in this study, the extensive studies at various pH will be useful to further understand the detailed response of Fe(II) oxidation to the HS chemical quality.

**Reference**

Pham, A.N., Waite, T.D., 2008. Oxygenation of Fe(II) in natural waters revisited: Kinetic modeling approaches, rate constant estimation and the importance of various reaction pathways. Geochim. Cosmochim. Acta 72, 3616-3630.
