# Supplementary material for: Variation of iron redox kinetics and its relation with molecular composition of standard humic substances at circumneutral pH
Source: PLoS One. 2017 Apr 28;12(4):e0176484. doi: 10.1371/journal.pone.0176484 (PMC5409151; doi:10.1371/journal.pone.0176484)
Supplement: S2 File — (DOCX) [file pone.0176484.s004.docx]

**S2 File. Inner filter effect in the photochemical experiment**

We examined the inner filter effect in the photochemical experiment by using the representative standard humic substance (i.e., SRFA). First, the UV-VIS absorbance spectrum for the SRFA from 300 nm to 700 nm was collected by using UV-VIS spectrophotometer (S2 Fig). Then, the inner filter effect due to the light absorbance by SRFA under the condition identical to that employed in the photochemical experiment was examined. As a result, percentages of light absorbed by SRFA in the 1 cm path-length cuvette were calculated to vary depending on the wavelength range of light ranging from 1.5% for 600 nm – 700 nm to 81% for 300 nm – 400 nm (S3 Table). While it was necessary to use higher HS concentration (200 mg/L) in the reduction experiment in order to ensure the insignificant competition of Fe(III) precipitation with Fe complexation by HS at circumneutral pH in this study, the calculation indicates that the concentration of HS employed in this study resulted in the significant light absorbance in the UV region. Although the degree of this inner filter effect on the photochemical reduction rate could not be extensively investigated in the present study, the development of experimental and analytical systems which allow us to use the lower humic substance concentration (e.g., novel technique to determine reduction rate of organically complexed Fe(III) with higher sensitivity) will be inevitable to quantify the photo-reduction rate in the absence of inner filter effect.
