# Supplementary material for: Variation of iron redox kinetics and its relation with molecular composition of standard humic substances at circumneutral pH
Source: PLoS One. 2017 Apr 28;12(4):e0176484. doi: 10.1371/journal.pone.0176484 (PMC5409151; doi:10.1371/journal.pone.0176484)
Supplement: S1 Table — (DOCX) [file pone.0176484.s005.docx]

**S1 Table. Molecular properties of standard humic substances used in this study.**

| Fraction | Code | Origin | Distribution source*^a^* | Elememtal ratio to carbon (mol mol^-1^) | | | | Carbon species estimated from ^13^C-NMR (%)*^b^* | | | | | | | | |
| --- | --- | --- | --- | --- | --- | --- | --- | --- | --- | --- | --- | --- | --- | --- | --- | --- |
|  |  |  |  |  |  |  |  | Aliphatic | Carbohydrate | | |  | Aromatic | Carbonyl | | |
|  |  |  |  | H/C | O/C | N/C | S/C |  | Heteroaliphatic | Acetal | Total |  |  | Carboxyl | Carbonyl | Total |
| HA | 2S101H*^f^* | Suwannee River II | IHSS | 0.970 | 0.600 | 0.0191 | 0.0039 | 29 | 13 | 7 | 20 |  | 31 | 15 | 6 | 21 |
|  | 1S102H*^f^* | Elliot Soil | IHSS | 0.754 | 0.440 | 0.0612 | 0.0029 | 16 | 6 | 4 | 10 |  | 50 | 18 | 6 | 24 |
|  | 1S103H*^f^* | Pahokee Peat | IHSS | 0.806 | 0.496 | 0.0560 | 0.0047 | 19 | 5 | 4 | 9 |  | 47 | 20 | 5 | 25 |
|  | 1S104H*^f^* | Leonardite | IHSS | 0.691 | 0.369 | 0.0165 | 0.0045 | 14 | 1 | 4 | 5 |  | 58 | 15 | 8 | 23 |
|  | 1R103H*^f^* | Pahokee Peat | IHSS | 0.755 | 0.484 | 0.0564 | 0.0047 | (n.a.) | (n.a.) | (n.a.) | (n.a.) |  | (n.a.) | (n.a.) | (n.a.) | (n.a.) |
|  | 1R105H*^f^* | Nordic Lake | IHSS | 0.887 | 0.606 | 0.0186 | 0.0041 | 15 | 11 | 7 | 18 |  | 38 | 19 | 10 | 29 |
|  | 1R107H*^f^* | Waskish Peat | IHSS | 0.881 | 0.530 | 0.0231 | 0.0024 | 18 | 8 | 6 | 14 |  | 42 | 18 | 8 | 26 |
|  | DHA*^g^* | Dando Soil | JHSS | 1.18 | 0.524 | 0.0726 | 0.0020 | 30.3 | (n.a.) | (n.a.) | 23.2 |  | 32.9 | (n.a.) | (n.a.) | 13.6 |
|  | IHA*^g^* | Inogashira Soil | JHSS | 0.930 | 0.502 | 0.0627 | 0.0018 | 22.3 | (n.a.) | (n.a.) | 22.2 |  | 36.5 | (n.a.) | (n.a.) | 19 |
| FA | 1S101F*^f^* | Suwannee River I | IHSS | 0.982 | 0.606 | 0.012 | 0.0032 | 33 | 11 | 5 | 16 |  | 24 | 20 | 7 | 27 |
|  | 2S101F*^f^* | Suwannee River II | IHSS | 0.995 | 0.618 | 0.011 | 0.0032 | 35 | 16 | 6 | 22 |  | 22 | 17 | 5 | 22 |
|  | 1R105F*^f^* | Nordic Lake | IHSS | 0.908 | 0.648 | 0.011 | 0.0032 | 18 | 12 | 7 | 19 |  | 31 | 24 | 10 | 34 |
|  | 1R109F*^f^* | Pony Lake | IHSS | 1.22 | 0.449 | 0.106 | 0.0216 | 61 | 8.4 | 0.2 | 8.6 |  | 12 | 17 | 1.2 | 18 |
|  | DFA*^g^* | Dando Soil | JHSS | 0.879 | 0.760 | 0.014 | 8 × 10^-5^ | 22.0 | (n.a.) | (n.a.) | 18.8 |  | 37.0 | (n.a.) | (n.a.) | 22.2 |
|  | IFA*^g^* | Inogashira Soil | JHSS | 0.953 | 0.889 | 0.0341 | 6 × 10^-4^ | 26.3 | (n.a.) | (n.a.) | 16.3 |  | 27.1 | (n.a.) | (n.a.) | 30.5 |
|  | BFA*^h^* | Lake Biwa | JHSS | 1.29 | 0.475 | 0.0353 | (n.a.) | 39.4 | (n.a.) | (n.a.) | 23.5 |  | 17.1 | 14.3 | 5.6 | 19.9 |

(n.a.): no data or not applicable.

*^a^* IHSS: International Humic Substances Society; JHSS: Japanese Humic Substances Society.

*^b^* Chemical shift of each species is as follows: 0–60 ppm (10–60 ppm for DHA, IHA, DFA and IFA, and 5–45 ppm for BFA) for aliphatic carbon, 60–90 ppm for heteroaliphatic carbon, 90–110 ppm for acetal carbon, 45–110 ppm for carbohydrate carbon of BFA, 110–165 ppm for aromatic carbon, 165–190 ppm for carboxyl carbon, 190–220 ppm for carbonyl carbon and 165–216 ppm for total carbonyl carbon of DHA, IHA, DFA and IFA.

**S1 Table.** Continued.

| Fraction | Code | Origin | Distribution source*^a^* | Aromaticity*^c^* | SUVA*^e^*  (L.mgC^-1^.m^-1^) | Acid functional groups (meq.gC^-1^) | |  | Total amino acids | Free radical |
| --- | --- | --- | --- | --- | --- | --- | --- | --- | --- | --- |
|  |  |  |  |  |  |  |  |  |  |  |
|  |  |  |  |  |  | Carboxyl*^d^* | Phenolic | | (μmol.gC^-1^) | (× 10^17^ spins.gC^-1^) |
| HA | 2S101H*^f^* | Suwannee River II | IHSS | 0.39 | 5.33 | 9.13 | 3.72 |  | (n.a.) | (n.a.) |
|  | 1S102H*^f^* | Elliot Soil | IHSS | 0.66 | 6.60 | 8.28 | 1.87 |  | 1337 | 22.2 |
|  | 1S103H*^f^* | Pahokee Peat | IHSS | 0.63 | 6.35 | 9.01 | 1.91 |  | 661 | 6.95 |
|  | 1S104H*^f^* | Leonardite | IHSS | 0.75 | 7.04 | 7.46 | 2.31 |  | 17 | 4.91 |
|  | 1R103H*^f^* | Pahokee Peat | IHSS | (n.a.) | 6.10 | 8.87 | 2.05 |  | 634 | 4.96 |
|  | 1R105H*^f^* | Nordic Lake | IHSS | 0.54 | 5.48 | 9.06 | 3.23 |  | 210 | 2.18 |
|  | 1R107H*^f^* | Waskish Peat | IHSS | 0.57 | 5.66 | (n.a.) | (n.a.) |  | (n.a.) | (n.a.) |
|  | DHA*^g^* | Dando Soil | JHSS | 0.381 | 4.60 | 6.98 | 4.11 |  | (n.a.) | (n.a.) |
|  | IHA*^g^* | Inogashira Soil | JHSS | 0.451 | 6.90 | 7.94 | 3.58 |  | (n.a.) | (n.a.) |
| FA | 1S101F*^f^* | Suwannee River I | IHSS | 0.33 | 3.89 | 11.44 | 2.91 |  | 46 | 1.0 |
|  | 2S101F*^f^* | Suwannee River II | IHSS | 0.28 | 4.64 | 11.17 | 2.84 |  | (n.a.) | (n.a.) |
|  | 1R105F*^f^* | Nordic Lake | IHSS | 0.46 | 4.75 | 11.16 | 3.18 |  | 69 | 2.18 |
|  | 1R109F*^f^* | Pony Lake | IHSS | 0.15 | 2.61 | (n.a.) | (n.a.) |  | (n.a.) | (n.a.) |
|  | DFA*^g^* | Dando Soil | JHSS | 0.476 | 4.52 | 16.2 | 2.86 |  | (n.a.) | (n.a.) |
|  | IFA*^g^* | Inogashira Soil | JHSS | 0.389 | 3.63 | 22.7 | 2.47 |  | (n.a.) | (n.a.) |
|  | BFA*^h^* | Lake Biwa | JHSS | 0.214 | 2.23 | (n.a.) | (n.a.) |  | (n.a.) | (n.a.) |

*^c^* Aromatic carbon (%) normalized to the sum of aliphatic, carbohydrate and aromatic carbon (%).

*^d^* Identical to the charge density of carboxyl functional group at pH 8.0 for the IHSS samples.

*^e^* Specific UV absorbance (SUVA) defined by absorbance at 254 nm relative to organic carbon concentration.

*^f^* The molecular properties listed have been determined and those data are provided by IHSS [40] (the reference number is identical to that in text).

*^g^* The molecular properties listed were determined by Watanabe et al. [38] (the reference number is identical to that in text).

*^h^* The molecular properties listed were determined by Fujitake et al. [39] (the reference number is identical to that in text).
