# Supplementary material for: Variation of iron redox kinetics and its relation with molecular composition of standard humic substances at circumneutral pH
Source: PLoS One. 2017 Apr 28;12(4):e0176484. doi: 10.1371/journal.pone.0176484 (PMC5409151; doi:10.1371/journal.pone.0176484)
Supplement: S2 Table — (DOCX) [file pone.0176484.s006.docx]

**S2 Table. Correlation coefficients (*r*) of iron (Fe) redox rate constants and steady-state Fe(II) fraction obtained in the presence of standard HS with the molecular parameters of HS.***^a^*

|  |  |  | Elememtal ratio to carbon (mol.mol^-1^) | | | | Carbon species estimated from ^13^C-NMR (%) | | | | | | | |
| --- | --- | --- | --- | --- | --- | --- | --- | --- | --- | --- | --- | --- | --- | --- |
|  |  |  | H/C | O/C | N/C | S/C | Aliphatic | Carbohydrate | | | Aromatic | Carbonyl | | |
|  |  |  |  |  |  |  |  | Heteroaliphatic | Acetal | Total |  | Carboxyl | Carbonyl | Total |
| k_O2_ | pH 8.0 | *n^b^* | 16 | 16 | 16 | 15 | 15 | 10 | 10 | 15 | 15 | 11 | 11 | 15 |
| (M^-1^ s^-1^) |  | *r* | **0.65^**^** | 0.20 | 0.061 | *0.064* | **0.54^*^** | **0.75^*^** | 0.096 | **0.54^*^** | **-0.66^**^** | 0.0023 | -0.25 | -0.257 |
|  | pH 7.0 | *n^b^* | 16 | 16 | 16 | 15 | 15 | 10 | 10 | 15 | 15 | 11 | 11 | 15 |
|  |  | *r* | -0.38 | -0.31 | 0.037 | *0.037* | **-0.55^*^** | -0.28 | 0.52 | 0.039 | **0.58^*^** | 0.34 | 0.57 | -0.14 |
| k_H2O2_ | pH 8.0 | *n^b^* | 16 | 16 | 16 | 15 | 15 | 10 | 10 | 15 | 15 | 11 | 11 | 15 |
| (× 10^3^ M^-1^ s^-1^) |  | *r* | **0.79^***^** | 0.092 | 0.074 | *-0.18* | **0.80^***^** | 0.48 | -0.37 | 0.27 | **-0.82^***^** | -0.38 | -0.56 | -0.25 |
|  | pH 7.0 | *n^b^* | 16 | 16 | 16 | 15 | 15 | 10 | 10 | 15 | 15 | 11 | 11 | 15 |
|  |  | *r* | **0.62^*^** | **0.51^*^** | -0.21 | *-0.045* | **0.61^*^** | **0.68^*^** | -0.015 | 0.26 | **-0.74^**^** | -0.21 | -0.34 | 0.065 |
| k_red_d_ | pH 8.0 | *n^b^* | 12 | 12 | 12 | 12 | 11 | 9 | 9 | 11 | 11 | 9 | 9 | 11 |
| (× 10^-6^ s^-1^) |  | *r* | 0.31 | **0.64^*^** | **-0.66^*^** | -0.020 | 0.50 | 0.56 | 0.27 | 0.28 | **-0.68^*^** | 0.46 | 0.12 | 0.44 |
|  | pH 7.0 | *n^b^* | 11 | 11 | 11 | 11 | 11 | 9 | 9 | 11 | 11 | 9 | 9 | 11 |
|  |  | *r* | -0.028 | 0.41 | -0.50 | -0.11 | 0.20 | 0.55 | 0.32 | 0.20 | -0.44 | 0.22 | 0.23 | 0.41 |
| k_red_p_ | pH 8.0 | *n^b^* | 11 | 11 | 11 | 11 | 11 | 9 | 9 | 11 | 11 | 9 | 9 | 11 |
| (× 10^-5^ s^-1^) |  | *r* | -0.30 | -0.40 | 0.049 | 0.017 | 0.0087 | -0.25 | -0.57 | -0.39 | 0.23 | -0.25 | -0.28 | -0.065 |
|  | pH 7.0 | *n^b^* | 11 | 11 | 11 | 11 | 11 | 9 | 9 | 11 | 11 | 9 | 9 | 11 |
|  |  | *r* | **-0.71^*^** | -0.51 | -0.15 | 0.39 | -0.32 | -0.51 | -0.62 | **-0.63^*^** | 0.47 | -0.25 | -0.12 | 0.19 |
| [Fe(II)]_ss_/[Fe]_T_, | pH 8.0 | *n^b^* | 12 | 12 | 12 | 12 | 11 | 9 | 9 | 11 | 11 | 9 | 9 | 11 |
| dark (%) |  | *r* | -0.31 | 0.027 | -0.45 | 0.19 | -0.085 | -0.23 | -0.37 | -0.40 | 0.012 | 0.41 | 0.19 | 0.56 |
|  | pH 7.0 | *n^b^* | 11 | 11 | 11 | 11 | 11 | 9 | 9 | 11 | 11 | 9 | 9 | 11 |
|  |  | *r* | -0.022 | 0.43 | -0.56 | 0.027 | 0.37 | 0.56 | 0.13 | 0.14 | -0.51 | 0.14 | -0.042 | 0.39 |
| [Fe(II)]_ss_/[Fe]_T_, | pH 8.0 | *n^b^* | 11 | 11 | 11 | 11 | 11 | 9 | 9 | 11 | 11 | 9 | 9 | 11 |
| light (%) |  | *r* | -0.51 | -0.56 | 0.19 | 0.047 | -0.28 | -0.46 | -0.63 | -0.55 | 0.48 | -0.21 | -0.20 | 0.0059 |
|  | pH 7.0 | *n^b^* | 11 | 11 | 11 | 11 | 11 | 9 | 9 | 11 | 11 | 9 | 9 | 11 |
|  |  | *r* | -0.51 | -0.17 | -0.32 | 0.35 | -0.030 | -0.089 | -0.44 | -0.40 | 0.12 | -0.16 | -0.26 | 0.28 |

**S2 Table.** Continued.

|  |  |  | Aromaticity | SUVA  (L.mgC^-1^.m^-1^) | Acid functional groups (meq.gC^-1^) | | Total amino acids | Free radical |
| --- | --- | --- | --- | --- | --- | --- | --- | --- |
|  |  |  |  |  |  |  |  |  |
|  |  |  |  |  | Carboxyl | Phenolic | (μmol.gC^-1^) | (× 10^17^ spins.gC^-1^) |
| k_O2_ | pH 8.0 | *n^b^* | 15 | 15 | 13 | 13 | 7 | 7 |
| (M^-1^ s^-1^) |  | *r* | **-0.68^**^** | **-0.53^**^** | 0.0503 | 0.55 | -0.63 | -0.70 |
|  | pH 7.0 | *n^b^* | 15 | 15 | 13 | 13 | 7 | 7 |
|  |  | *r* | **0.53^*^** | **0.70^**^** | **-0.80^***^** | 0.38 | 0.044 | -0.23 |
| k_H2O2_ | pH 8.0 | *n^b^* | 15 | 15 | 13 | 13 | 7 | 7 |
| (× 10^3^ M^-1^ s^-1^) |  | *r* | **-0.82^***^** | **-0.86^***^** | 0.55 | 0.27 | -0.52 | -0.63 |
|  | pH 7.0 | *n^b^* | 15 | 15 | 13 | 13 | 7 | 7 |
|  |  | *r* | **-0.70^**^** | **-0.87^***^** | **0.64^*^** | 0.25 | **-0.80^*^** | **-0.87^*^** |
| k_red_d_ | pH 8.0 | *n^b^* | 11 | 11 | 11 | 11 | 7 | 7 |
| (× 10^-6^ s^-1^) |  | *r* | -0.60 | **-0.69^*^** | **0.87^***^** | 0.17 | -0.72 | -0.65 |
|  | pH 7.0 | *n^b^* | 11 | 11 | 10 | 10 | 6 | 6 |
|  |  | *r* | -0.36 | -0.23 | 0.60 | 0.038 | -0.46 | -0.48 |
| k_red_p_ | pH 8.0 | *n^b^* | 11 | 11 | 10 | 10 | 6 | 6 |
| (× 10^-5^ s^-1^) |  | *r* | 0.21 | 0.074 | -0.032 | -0.52 | 0.53 | 0.74 |
|  | pH 7.0 | *n^b^* | 11 | 11 | 10 | 10 | 6 | 6 |
|  |  | *r* | 0.48 | 0.44 | -0.030 | **-0.70^*^** | 0.25 | 0.54 |
| [Fe(II)]_ss_/[Fe]_T_, | pH 8.0 | *n^b^* | 11 | 11 | 11 | 11 | 7 | 7 |
| dark (%) |  | *r* | 0.10 | -0.094 | 0.50 | -0.31 | -0.57 | -0.37 |
|  | pH 7.0 | *n^b^* | 11 | 11 | 10 | 10 | 6 | 6 |
|  |  | *r* | -0.43 | -0.37 | **0.75^*^** | -0.13 | -0.30 | -0.28 |
| [Fe(II)]_ss_/[Fe]_T_, | pH 8.0 | *n^b^* | 11 | 11 | 10 | 10 | 6 | 6 |
| light (%) |  | *r* | 0.46 | 0.34 | -0.20 | -0.61 | 0.68 | **0.86^*^** |
|  | pH 7.0 | *n^b^* | 11 | 11 | 10 | 10 | 6 | 6 |
|  |  | *r* | 0.15 | 0.12 | 0.35 | **-0.66^*^** | 0.34 | 0.60 |

*^a^* Spearman's rank correlation coefficient was calculated and indicated as italic letters if either or both of the parameter sets of interest was non-normally distributed, otherwise Pearson's product-moment correlation coefficient was calculated. Bold-faced numbers indicate that the correlations are statistically significant (^*^: *p* < 0.05; ^**^: *p* < 0.01; ^***^: *p* < 0.001).

*^b^* Number of data pair.
