# Supplementary material for: Variation of iron redox kinetics and its relation with molecular composition of standard humic substances at circumneutral pH
Source: PLoS One. 2017 Apr 28;12(4):e0176484. doi: 10.1371/journal.pone.0176484 (PMC5409151; doi:10.1371/journal.pone.0176484)
Supplement: S3 Table — (DOCX) [file pone.0176484.s007.docx]

**S3 Table. The light absorbed by SRFA in the photochemical experiment**

| Parameter | Symbol | Unit | Wavelength range | | | |
| --- | --- | --- | --- | --- | --- | --- |
|  |  |  | 300 nm - 400 nm | 400 nm - 500 nm | 500 nm - 600 nm | 600 nm - 700 nm |
| SRFA concentration | *c* | mg/L | 200 | 200 | 200 | 200 |
| Average molar absorptivity | *ε* | [mg/L]^-1^.m^-1^ | 0.357 | 0.093 | 0.019 | 0.003 |
| Length of light path*^a)^* | *l* | cm | 1.0 | 1.0 | 1.0 | 1.0 |
| Absorbance | *A =εcl* | cm^-1^ | 0.713 | 0.185 | 0.038 | 0.007 |
| Light absorbed | *I_a_/I_0_ = (1-10^-εcl^)* |  | 0.807 | 0.347 | 0.084 | 0.015 |
